# *In vivo* Magnetic Resonance Microscopy and Hypothermic Anaesthesia of a Disease Model in Medaka

**DOI:** 10.1038/srep27188

**Published:** 2016-06-02

**Authors:** Tomohiro Ueno, Hirokazu Suzuki, Masahiro Hiraishi, Hideaki Amano, Hidenao Fukuyama, Naozo Sugimoto

**Affiliations:** 1 Human Health Sciences, Graduate School of Medicine, Kyoto University, Kyoto 606-8507, Japan; 2 Human Health Sciences, Faculty of Medicine, Kyoto University, Kyoto 606-8507, Japan; 3 Human Brain Research Center, Graduate School of Medicine, Kyoto University, Kyoto 606-8507, Japan

## Abstract

In medical and pharmacological research, various human disease models in small fish, such as medaka (*Oryzias latipes*), have been created. To investigate these disease models noninvasively, magnetic resonance imaging (MRI) is suitable because these small fish are no longer transparent as adults. However, their small body size requires a high spatial resolution, and a water pool should be avoided to maximize the strength of MRI. We developed *in vivo* magnetic resonance microscopy (MR microscopy) without a water pool by combining hypothermic anaesthesia and a 14.1 T MR microscope. Using *in vivo* MR microscopy, we noninvasively evaluated the hepatic steatosis level of a non-alcoholic fatty liver disease model in medaka and followed the individual disease progression. The steatosis level was quantified by the MRI-estimated proton density fat-fraction (MRI-PDFF), which estimates the triglyceride fat concentration in liver tissue and is recognized as an imaging biomarker. The MRI-PDFF results agreed with a histological analysis. Moreover, we optimized the hypothermic anaesthesia procedure to obtain a recovery proportion of 1 in the experiment involving MR microscopy. Recovered medaka could not be distinguished from naïve medaka after the experiment. Therefore, the *in vivo* MR microscopy will expand the possibilities of a human disease model in fish.

Medaka (*Oryzias latipes*) is a small egg-laying freshwater fish that is very hardy and tolerates a wide range of temperatures. Because their eggs and embryos are transparent, medaka—along with zebrafish (*Danio rerio*) —are used as an excellent vertebrate animal model for developmental biology and genetics^1^. Furthermore, due to progress in genome-editing techniques, various human disease models in medaka, such as a tumour suppressor gene *P53* knockout model^2^ and a neuronopathic Gaucher disease model^3^, have been developed. In addition to gene-manipulated disease models, a model for non-alcoholic steatohepatitis, a progressive form of non-alcoholic fatty liver disease (NAFLD), can be created simply by feeding medaka with a high-fat diet (HFD) because medaka and humans share metabolic similarities^4^. Although there are several inbred medaka strains and genetic similarities between those strains, these diseases occur and develop heterogeneously in each fish^2^,^3^,^4^. Moreover, these diseases develop at a later stage of life, when medaka are no longer transparent. However, a histological study cannot follow individual disease progression in each fish.

Magnetic Resonance Imaging (MRI) is a non-invasive diagnostic imaging technique. Using MRI, we can visualize spatial distributions of various physical and chemical properties of internal organs. For example, we can separate a fat component from a water component in an MRI image^5^. Moreover, we can evaluate hepatic steatosis with an MRI-estimated proton density fat-fraction (MRI-PDFF)^6^,^7^. The PDFF has been found to highly correlate with biochemically measured triglyceride fat concentrations in tissues and is considered as an MR-based biomarker of tissue fat concentration^6^. Moreover, a longitudinal study has shown that the MRI-PDFF is more sensitive than histological grading of steatosis in quantifying changes in the fat concentration in human livers^7^. Although the cell size of medaka is similar to that of humans, a high spatial resolution of MRI is required to investigate a disease model in medaka. Because MRI basically visualizes a water component, a water pool of fish generates strong background signals and should be avoided. A high-resolution MRI, the MR microscope, has been developed by using a 14.1 T magnetic field and 3 T/m magnetic field gradients^8^. This technique was used to visualize *ex vivo P53* knockout medaka with an isotropic 20 μm voxel without a water pool. However, the imaging time was too long (20 hours) for an *in vivo* study. Using a faster MRI sequence and a coarser pixel size (78 μm × 78 μm, slice thickness 0.2 mm), an *in vivo* MRI of a disease model in zebrafish in a water pool has been conducted^9^. In the MRI of zebrafish, anaesthetic water flowed through fish to deliver oxygen. The water flow near the fish, however, causes vibrations and limits the applicable MRI sequences. Although anaesthetic water can reduce the breathing rate of fish, it cannot completely stop breathing motions, which also cause body vibrations; moreover, the anaesthetic has a high probability of stopping the heart from beating^10^. As long as the breathing motions exist, oxygen must be delivered by water flow.

Fish are known to stop breathing and to achieve an anaesthetized state when immersed in very cold water. This hypothermia induces light anaesthesia and is known to be useful for transport^11^. In this study, by combining the 14.1 T MR microscope and hypothermic anaesthesia, we developed *in vivo* MR microscopy without a water pool and applied it to an NAFLD model in medaka. We calculated the MRI-PDFF of a liver and evaluated the progression of hepatic steatosis in a group of medaka and in individual medaka. We compared the MRI-PDFF results with a histological analysis and obtained reasonable agreement. Furthermore, by optimizing the hypothermic anaesthesia procedure, we obtained a recovery proportion of 1 for the disease model in the MR microscopy experiments.

## Results

### Lowering water temperature stops opercular movements, but not heartbeats

**Figure 1:**
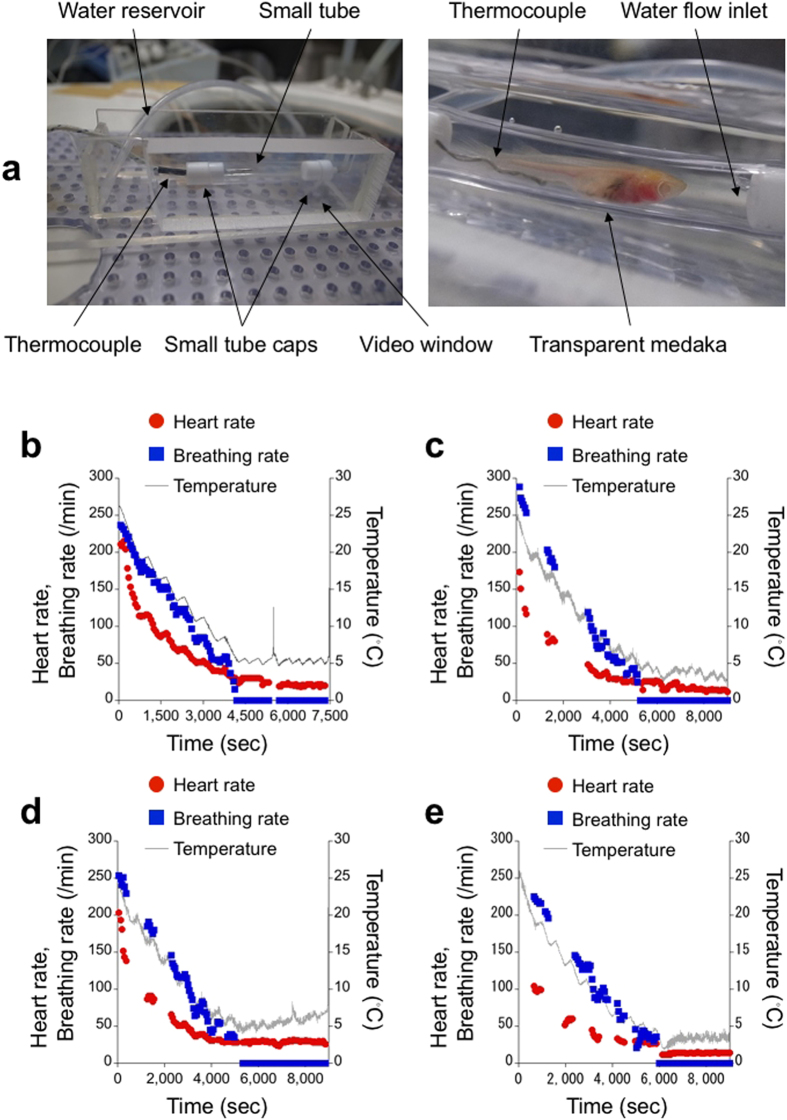
Hypothermic anaesthesia leads to cessation of opercular movements of medaka but maintains heartbeats. (**a**) A medaka chamber used in the hypothermic anaesthesia experiment. The water temperature was measured using a thermocouple near the transparent medaka (STIII). Heartbeats and opercular movements were videotaped from the side of the chamber. (**b**) The heart rate and breathing rate are correlated with water temperature. At approximately 5 °C, the breathing rate dropped to zero, but the heart rate did not. With or without water in a tube, heartbeats existed and opercular movements ceased. Sudden temperature increases at 5,500 and 7,484 seconds corresponded to the draining of water and returning the transparent medaka to a tank, respectively. (**c**) During the 60 minutes in which the opercular movements were lost, the heart rate decreased slightly with a temperature decrease. (**d**) Although the temperature increased during the 60 minutes, the heart rate stayed constant. (**e**) In the case of constant temperature during the 60 minutes, the heart rate also stayed constant. A sudden change in the temperature at the beginning of the 60 minutes decreased the constant heart rate. All transparent medaka in (**b**–**e**) recovered uneventfully after the experiments.

To infer a physiological state during the imaging time of the MR microscope, we investigated the physiological changes in medaka due to the hypothermic anaesthesia. We videotaped transparent medaka^12^ in a small tube as the water temperature in the tube was lowered, and we counted heartbeats and opercular movements (Fig. 1a). First, we limited the duration of stage 3 of the hypothermic anaesthesia to 20 minutes and then drained the water from the small tube while maintaining the temperature inside of the tube (Fig. 1b). Here, stage 3 anaesthesia corresponds to a stage in which the gross body movements and opercular movements of the fish are lost^13^. The temperature at which the transparent medaka entered stage 3 was 5.4 °C. After we drained the water, we kept the transparent medaka in the small tube at stage 3 for 30 minutes. The transparent medaka recovered uneventfully after a total of 50 minutes at stage 3. Afterwards, the transparent medaka lived for two months. We found that the heart rate and the breathing rate were correlated with temperature. This finding corresponds to the temperature characteristics of physiological rhythmic phenomena^14^,^15^,^16^,^17^. Moreover, even after the opercular movements ceased, the heart kept beating, though its correlation with temperature did not seem strong.

By removing disturbances caused by the draining water, we extended the duration of stage 3 to 60 minutes with a slight variation in the water temperature. We used 9 naïve, transparent medaka (6 males, 3 females; mean body length 21 mm). After 60 minutes of the stage 3 of hypothermic anaesthesia without an external oxygen supply, all transparent medaka survived. However, 3 transparent medaka, which did not start opercular movements even two hours later, were confirmed dead on the next day of the experiment. The remaining 6 transparent medaka, whose opercular movements were confirmed within two hours after the experiment, lived for more than two weeks and showed no differences in behaviour from naïve, transparent medaka during the two-week observation period. The recovered transparent medaka followed the typical stages of recovery from anaesthesia^13^ (1: starting opercular movements without gross body movements; 2: starting gross body movements with regular opercular movements; 3: regaining equilibrium), although each recovery time was different. We could not distinguish the recovered transparent medaka from the naïve transparent medaka in the next day of the experiment. Figure 1c–e shows the typical temperature dependences of heart rates. Even in the extended period of stage 3, the heart kept beating (Fig. 1c–e), although there were individual differences in the temperatures at which the opercular movements ceased and in the initial heart and breathing rates. When we lowered the water temperature, the heart rate decreased slightly with the temperature (Fig. 1c). By contrast, when we raised the temperature, the heart rate was constant (Fig. 1d). In the third case, we decreased the water temperature from 3.5 °C to 2.0 °C just after the opercular movements ceased. Then, we increased the temperature and kept it to 3.5 °C (Fig. 1e). The heart rate decreased with the water temperature and then recovered to a constant level. The recovered heart rate, however, was lower than the value just before the opercular movements ceased (Fig. 1e).

### Histological analysis shows steatosis in the medaka NAFLD model

**Figure 2:**
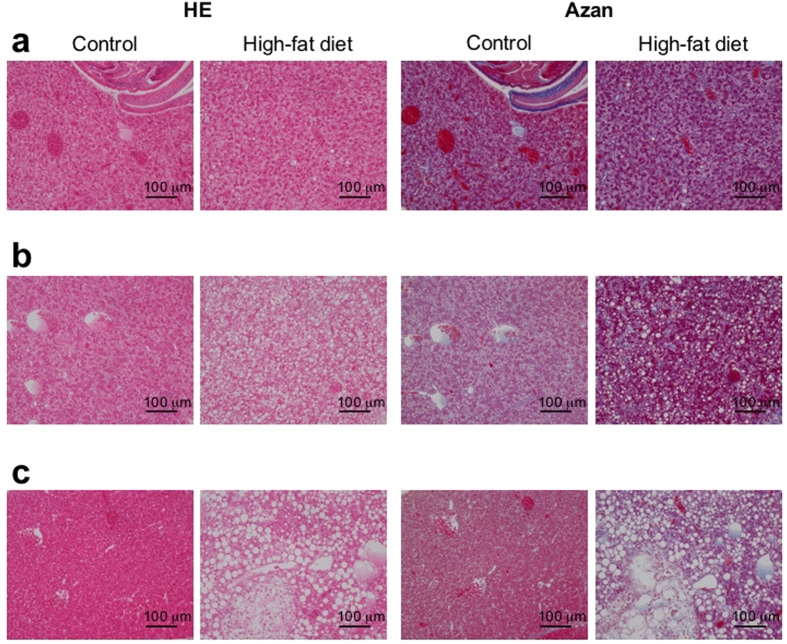
Feeding a high-fat diet induces hepatic steatosis in medaka. (**a**) Histological analysis in medaka (Kyoto-cab strain) fed an HFD for 6 weeks (right sub-column) and in medaka (Kyoto-cab strain) maintained under control conditions for 6 weeks (left sub-column) using haematoxylin and eosin (HE) staining (left column) and azan staining (right column). Scale bars represent 100 μm. (**b**) Histological analysis in medaka (Kyoto-cab strain) of the 12 weeks HFD period (right sub-column) and in medaka (Kyoto-cab strain) maintained under control conditions for 12 weeks (left sub-column) using HE staining (left column) and azan staining (right column). The HFD group showed severe steatosis and hepatocellular ballooning. The azan staining indicated fibrosis in the HFD group. Scale bars represent 100 μm. (**c**) Histological analysis in medaka (Kyoto-cab strain) of the 16 weeks HFD period (right sub-column) and in medaka (Kyoto-cab strain) maintained under control conditions for 16 weeks (left sub-column) using HE staining (left column) and azan staining (right column). HE staining revealed an improvement of steatosis in the control group. The HFD group showed a heterogeneous distribution of larger lipid droplets and hepatocellular ballooning. Scale bars represent 100 μm.

We carried out a histological analysis in the HFD group of wild-type medaka (*N* = 3; HFD periods = 6, 12, 16 weeks) and in a control group (*N* = 3; control periods = 6, 12, 16 weeks). The four following histological features of NAFLD can be semi-quantitatively evaluated using an NAFLD activity score^18^: steatosis, lobular inflammation, hepatocellular ballooning, and fibrosis. To examine these features, we used haematoxylin-eosin (HE) staining and azan staining, in which fibrosis is stained blue. After the HFD period of 6 weeks, slight steatosis was observed in an HE stained section of the HFD group (Fig. 2a). Even in a normal liver section of the control group, however, slight steatosis was observed (Fig. 2a). No features of fibrosis was observed in the azan-stained sections of either group after the feeding period of 6 weeks (Fig. 2a).

After the HFD period of 12 weeks, severe steatosis was observed in an HE stained section of the HFD group (Fig. 2b). Hepatocellular ballooning was also observed in many hepatocytes in the HFD group (Fig. 2b). After the control period of 12 weeks, slight steatosis was observed in the control group (Fig. 2b). The indication of fibrosis appeared in an azan-stained section of the HFD group, but not in the control group (Fig. 2b).

After 16 weeks of HFD, severe steatosis was also observed in an HE-stained section of the HFD group (Fig. 2c), and the lipid droplets were larger and more than those in the HE section after 12 weeks of HFD (Fig. 2b,c). In the control group, no steatosis was observed in an HE section of the control group (Fig. 2c). In the control group, the slight steatosis observed at the control period of 6 weeks was improved to a healthy state (Fig. 2a–c). There was no indication of fibrosis in the azan-stained sections of the two groups (Fig. 2c). The foci of lobular inflammation were seldom observed in the HE-stained sections for all periods (Fig. 2a–c).

### Overnight acclimation increases the proportion of uneventful recoveries of a medaka disease model in the MR microscopy experiment

**Figure 3:**
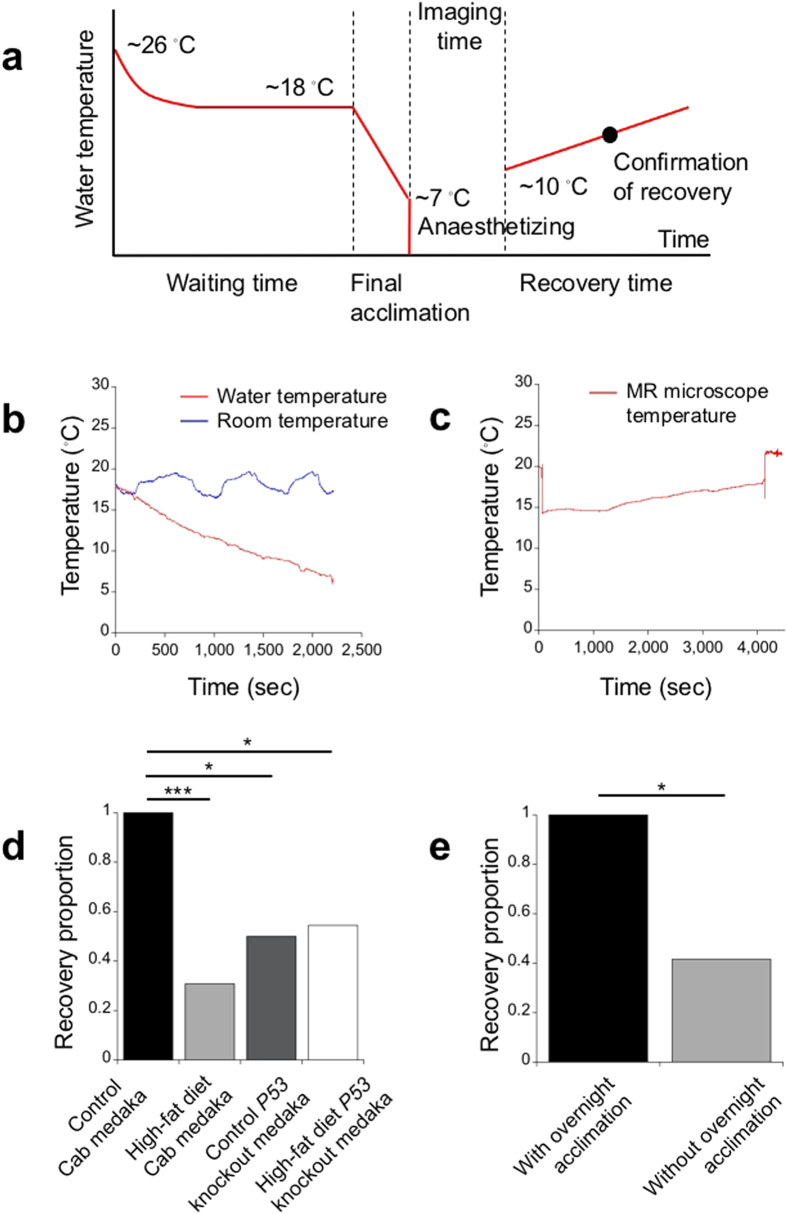
Adding an overnight acclimation period to the hypothermic anaesthesia procedure increases the recovery proportion of a medaka disease model in the MR microscopy experiment. (**a**) Typical water-temperature change during the entire hypothermic anaesthesia procedure. (**b**) Typical changes in water temperature (red) and room temperature (blue) in the final acclimation. (**c**) Typical temperature changes of the MR microscope during the imaging time. The temperature was measured just outside of the medaka imaging space near the gradient coils. The sudden changes at the beginning and the end correspond to the actions of placing the MR microscope probe in a magnet and pulling out the magnet, respectively. (**d**) The recovery proportion of the control Cab medaka was measured to be 1 and was significantly higher than that of the HFD Cab medaka, control *P53* knockout medaka and HFD *P53* knockout medaka in the MR microscopy experiment without the overnight acclimation (control Cab medaka: *N* = 18; HFD Cab medaka: *N* = 13; control *P53* knockout medaka: *N* = 6; HFD *P53* knockout medaka: *N* = 11). There was no statistical difference between the recovery proportions of the HFD Cab medaka, control *P53* knockout medaka and HFD *P53* knockout medaka. (**e**) Adding the overnight acclimation caused the combined recovery proportion of the HFD Cab medaka and the HFD *P53* knockout medaka to be 1 (*N*_w/overnight acclimation_ = 6; HFD Cab medaka: *N* = 3; HFD *P53* knockout medaka: *N* = 3), which was significantly higher than that of the HFD Cab medaka and the HFD *P53* knockout medaka without the overnight acclimation (*N*_w/o overnight acclimation_ = 24; HFD Cab medaka: *N* = 13; HFD *P53* knockout medaka: *N* = 11). Here, one-sided Fisher’s exact tests were used. ***P < 0.0001, *P < 0.05.

In performing the MR microscopy experiments, we applied hypothermic anaesthesia to the medaka. The entire procedure of the applied hypothermic anaesthesia consisted of the following 5 periods: waiting time, final acclimation, anaesthetizing, imaging time, and recovery time (Fig. 3a). During the waiting time, the medaka were brought into an MR microscope room with a room temperature of approximately 18 °C, and they waited to be visualized. We used two types of the waiting times, i.e., with or without overnight acclimation to the cooled room temperature. The waiting time without the overnight acclimation varied from 0 to 10 hours depending on the order of the MR microscopy experiment; the overnight acclimation added approximately 18 hours (Fig. 3a). Therefore, when the waiting time did not include the overnight acclimation, the starting water temperature of the final acclimation period was decreased gradually to the temperature of the cooled room as the experiment progressed. In the final acclimation, the water temperature was decreased to approximately 7 °C in 40 to 60 minutes (Fig. 3a,b). This cooling rate was similar to the one used for the transparent medaka. After the final acclimation, the medaka were anaesthetized in iced water (Fig. 3a). After opercular movements ceased, the medaka were taken out of the water and placed in the MR microscope without water (Fig. 3a). Although the MR microscope was cooled by refrigerated air, the temperature of next to the sample space increased gradually during the imaging time due to gradient coil heating (Fig. 3c). The imaging time was approximately 70 minutes (Fig. 3a,c). After the imaging time, medaka were returned to the water used in the final acclimation (Fig. 3a). In the medaka that recovered uneventfully, the typical recovery process was as follows. Upon being placed in the water, the medaka started opercular movements and increased their breathing rate gradually without swimming movements. In approximately 30 minutes, the medaka started swimming. Then, in approximately one hour, medaka regained equilibrium and swam uneventfully. When we confirmed that medaka swam uneventfully, we determined that the uneventful recovery of medaka was achieved. The uneventfully recovered medaka could not be distinguished from naïve medaka by their behaviour, and they lived for longer than a few months. On the other hand, the medaka that did not start opercular movements upon being placed in the water would not recover uneventfully.

Using the above hypothermic anaesthesia procedure, we performed MR microscopy experiments on 35 wild-type Cab medaka (Kyoto-cab strain), which consisted of 18 control Cab medaka and 17 HFD Cab medaka. In addition to the wild-type Cab medaka, we performed MR microscopy experiments on 20 *P53* knockout medaka (*P53*^*E241X/E241X*^)^2^, which consisted of 6 control *P53* knockout medaka and 14 HFD *P53* knockout medaka, using the same HFD feeding procedure and hypothermic anaesthesia procedure. However, we could create control *P53* knockout medaka and HFD *P53* knockout medaka only during limited feeding periods (control *P53* knockout medaka: *N*_12w_ = 6; HFD *P53* knockout medaka: *N*_12w_ = 6, *N*_17w_ = 1, *N*_19w_ = 4, *N*_22w_ = 3). In Table 1, we summarized a proportion of uneventful recoveries of the medaka after the MR microscopy experiment.

At first, we adopted the hypothermic anaesthesia procedure without the overnight acclimation. All 18 control Cab medaka (*N*_6w_, *N*_12w_, *N*_16w_ = 6) recovered uneventfully after the MR microscopy experiment. In contrast, only 4 out of 13 HFD Cab medaka (*N*_6w_, *N*_12w_ = 6; *N*_17w_ = 1) recovered uneventfully after the experiment. The uneventfully recovered HFD Cab medaka consisted of one HFD Cab medaka at 6 weeks and 3 HFD Cab medaka at 12 weeks. Here, we excluded from the total count one HFD Cab medaka at 17 weeks that experienced hypothermic anaesthesia two days before the experiment. The proportion of uneventful recoveries in the control Cab medaka was significantly higher (*P* < 0.0001, as determined by a one-sided Fisher’s exact test) than that of the HFD Cab medaka (Fig. 3d). As for the *P53* knockout medaka, 3 in 6 control *P53* knockout medaka (*N*_12w_ = 6) and 6 in 11 HFD *P53* knockout medaka (*N*_12w_ = 6, *N*_17w_ = 1, *N*_19w_ = 4) recovered uneventfully. The uneventfully recovered HFD *P53* knockout medaka included 3 HFD *P53* knockout medaka at 12 weeks and 3 HFD *P53* knockout medaka at 19 weeks. The proportion of uneventful recoveries of the control Cab medaka was significantly higher (*P* < 0.05) than that of the control *P53* knockout medaka and the HFD *P53* knockout medaka (Fig. 3d). There was no significant difference in the recovery proportions of the HFD Cab medaka, control *P53* knockout medaka or HFD *P53* knockout medaka (Fig. 3d). In the same day, we performed the MR microscopy experiment on 6 HFD Cab medaka at 12 weeks, and 3 uneventfully recovered HFD Cab medaka were visualized in the latter part of the experiment. This fact indicated that a longer period of acclimation to the cooled room temperature was preferable for the HFD group.

To increase the proportion of recovery for medaka disease models, we added an overnight acclimation. As a result, all 3 HFD Cab medaka at 16 weeks and all 3 HFD *P53* knockout medaka at 22 weeks recovered uneventfully after the MR microscopy experiment. The proportion of uneventful recoveries of the HFD groups with the overnight acclimation was significantly higher than that without the overnight acclimation (*P* < 0.05; Fig. 3e).

After the MR microscopy experiment, we sacrificed the medaka to confirm the condition of their internal organs. We could not find any difference between the uneventfully recovered medaka and non-recovered medaka. To investigate the long-term effects of the hypothermic anaesthesia procedure, we continued breeding some of uneventfully recovered medaka (control Cab medaka after 12 weeks. The quantity of medaka and groups are as follows: *N* = 6; control *P53* knockout medaka after 16 weeks: *N* = 1; HFD Cab medaka after 12 weeks: *N* = 2; after 16 weeks: *N* = 3; HFD *P53* knockout medaka after 12 weeks: *N* = 1; after 19 weeks: *N* = 2; and after 22 weeks: *N* = 2). Except for the medaka that were sacrified for histological analysis or non-recovered by 4 weeks after the second MR microscopy experiment (HFD Cab medaka at 12 weeks: *N* = 2; HFD *P53* knockout medaka at 12 weeks: *N* = 1), all medaka lived for more than 2 months after the MR microscopy experiment. With or without the overnight acclimation, the uneventfully recovered medaka could not be distinguished from naïve medaka by their behaviour.

### *In vivo* MR microscopy of medaka and its fat tissue-quantification analysis

**Figure 4:**
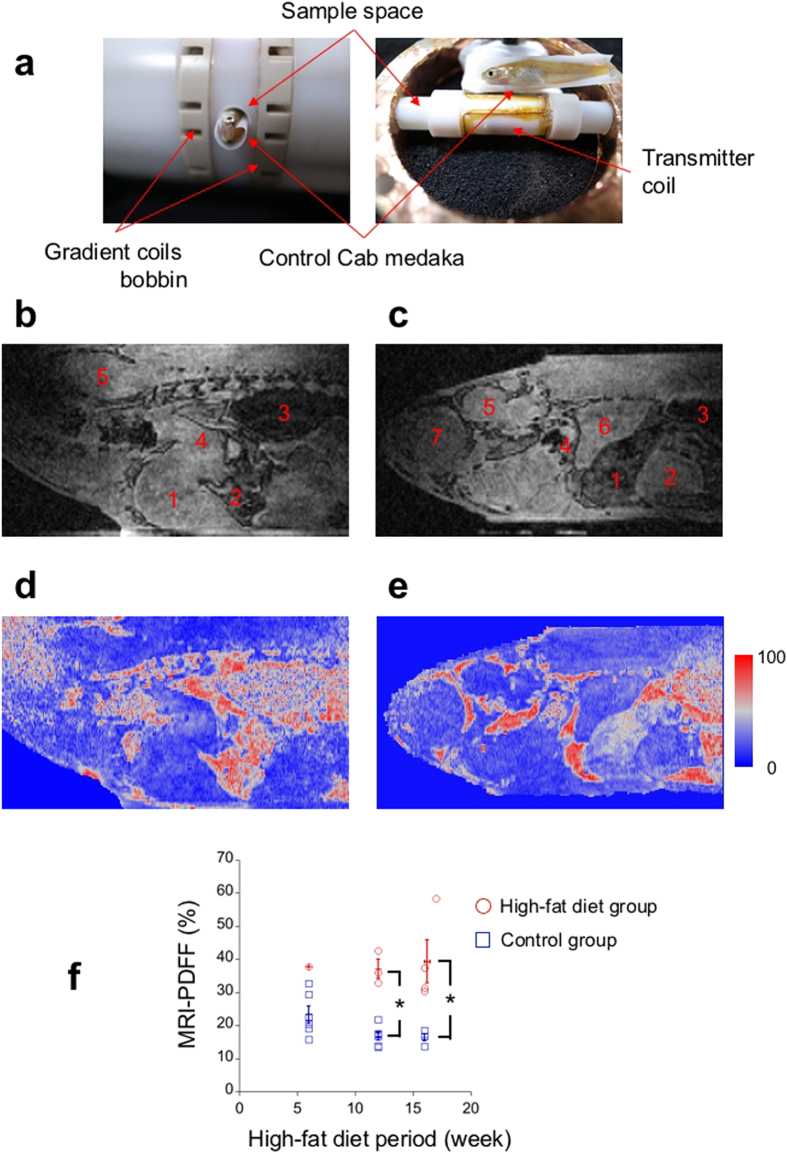
*In vivo* MR microscopy shows higher fat accumulation of the high-fat-diet group than of the control group. (**a**) The sample space of the MR microscope relative to the control Cab medaka at 12 weeks is shown (left: the control Cab medaka is inside of the sample space; right: the medaka is next to the transmitter coil and the sample space). Under the transmitter coil, the receiver coil is wound around the sample space. (**b**) The sagittal anatomical image of the control Cab medaka at 12 weeks shows the following internal organs: the liver (1); the gall bladder (2); the air bladder (3); the digestive tract (4); and the brain (5). (**c**) The sagittal anatomical image of the HFD Cab medaka at 12 weeks shows internal organs with different contrasts; the liver (1); the gall bladder (2); the air bladder (3); the digestive tract (4); the brain (5); the kidney (6); and the eye ball (7). (**d**) The MRI-PDFF map of the control Cab medaka at 12 weeks, which is at the same slice position as (**b**), shows relatively low intensity in the liver. (**e**) The MRI-PDFF map of the HFD Cab medaka at 12 weeks, which is at the same slice position as (**c**), shows higher intensity in the liver, particularly on the dorsal side. (**f**) At the feeding periods of 12 weeks and 16 weeks, the mean MRI-PDFFs of the livers of the HFD group are significantly higher than those of the control group (HFD Cab medaka: *N*_12w_ = 3, *N*_16w_ = 4; control Cab medaka: *N*_12w_ = 6, *N*_16w_ = 4; *P < 0.05). Here, two-sided Mann-Whitney tests were used. The data are presented as individual points superimposed by the mean ± s.e.m. The MR microscopy images were bicubically interpolated to have an isovoxel (34 μm).

Using the hypothermic anaesthesia procedure, we performed MR microscopy experiments on the control and the HFD groups of medaka. In an MR microscopy experiment for a single, individual medaka, we obtained three 3D gradient echo image datasets of voxel size 34 μm × 58 μm × 58 μm with different echo times (TE), which took approximately 70 minutes, including the image-adjustment time. Figure 4a shows a sample space of the MR microscope with a control Cab medaka at 12 weeks, which is different from the medaka in Fig. 4b–e. Because we could not obtain enough data for the control *P53* knockout or HFD *P53* knockout medaka, we analysed in detail only the MR microscope data for the control Cab and HFD Cab medaka. Figure 4b,c show typical sagittal slice images of a control Cab medaka at 12 weeks (Fig. 4b) and of an HFD Cab medaka at 12 weeks (Fig. 4c). Here, the sagittal slices were obtained from the 3D gradient echo medaka images with TE 1.150 ms that were bicubically interpolated to have an isovoxel size (34 μm). Both medaka in Fig. 4b–e recovered uneventfully after the MR microscopy experiments. In Fig. 4b,c, the internal organs of the medaka are visible and include the following: the liver (Fig. 4b,c1), the gall bladder (Fig. 4b,c2), the air bladder (Fig. 4b,c3), the digestive tract (Fig. 4b,c4), the brain (Fig. 4b,c5), a kidney (Fig. 4c6), and an eye ball (Fig. 4c7). Comparing the digestive organs, the liver of the HFD Cab medaka appears darker than that of the control Cab medaka (Fig. 4b,c). In addition, the gall bladder of the HFD Cab medaka is much larger than that of the control Cab medaka and is similar in size to the liver (Fig. 4b,c).

To quantify fat tissue in a liver, we calculated MRI-PDFF from three 3D MR microscope datasets with different TEs. The MRI-PDFF represents the ratio of a fat component to a total of a fat and a water component in a voxel. Figure 4d,e shows sagittal slice images of MRI-PDFF that correspond to the same slices in Fig. 4b,c. Comparing livers, the MRI-PDFF of the HFD Cab medaka shows a higher value than that of the control Cab medaka (Fig. 4d,e). Moreover, a more heterogeneous distribution of MRI-PDFF was found in the HFD Cab medaka (Fig. 4e). To obtain an individual indicator of fat tissue in a liver, we averaged the MRI-PDFF over a liver region of a selection of 2D slices. The mean values of the MRI-PDFF of the control Cab medaka (Fig. 4d) and of the HFD Cab medaka (Fig. 4e) were 16.8% and 32.9%, respectively.

We calculated the mean values of the MRI-PDFF of livers for the control Cab medaka group and the HFD Cab medaka group that recovered uneventfully after the MR microscopy experiments. In the entire HFD period, the HFD Cab medaka group showed a higher mean MRI-PDFF than the control Cab medaka group (Fig. 4f). We performed a two-sided Mann-Whitney test and found that the mean MRI-PDFFs of the HFD Cab medaka group were significantly higher after HFD for 12 weeks (HFD Cab medaka: *N*_12w_ = 3; *P* < 0.05) and 16 and 17 weeks (HFD Cab medaka: *N*_16w_ = 3, *N*_17w_ = 1; *P* < 0.05) than those of the control Cab medaka group (control Cab medaka: *N*_12w_ = 6, *N*_16w_ = 4). Here, we could not analyse two control Cab medaka at 16 weeks due to a striped pattern in their MRI-PDFF maps. Because only one HFD Cab medaka at 6 weeks recovered uneventfully, we could not perform a statistical analysis of the HFD period of 6 weeks.

### Individual time-series analysis of medaka with high-fat diets

Three HFD Cab medaka receiving HFD for 12 weeks recovered uneventfully after the MR microscopy experiments without the overnight acclimation. To investigate an individual time series, we restarted to feed an HFD to two of the three recovered HFD Cab medaka. The one remaining medaka was used for histological analysis. After an HFD period of 17 weeks, we performed MR microscopy experiments on the two uneventfully recovered HFD Cab medaka. Only one of them recovered uneventfully after the MR microscopy experiment without the overnight acclimation. Notably, the uneventfully recovered HFD Cab medaka experienced the hypothermic anaesthesia the two days before the MR microscopy experiment. We examined the time series of the twice uneventfully recovered HFD Cab medaka.

**Figure 5:**
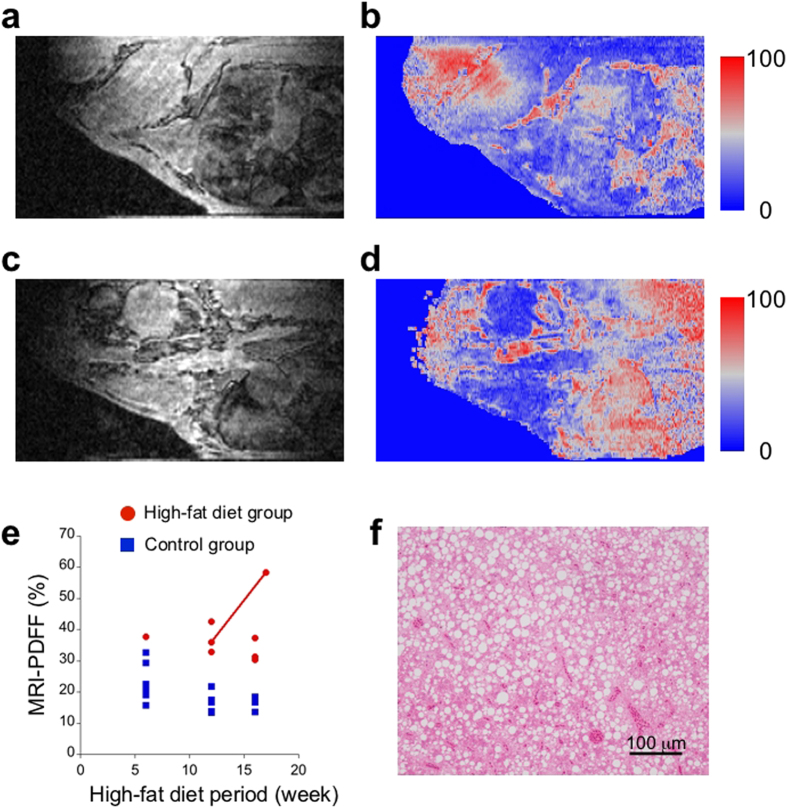
The time-series analysis of the same high-fat-diet Cab medaka shows an increase in the mean MRI-PDFF of the liver. (**a**) The anatomical image of the HFD Cab medaka at 12 weeks shows the large liver section at the centre. (**b**) The MRI-PDFF map of the HFD Cab medaka at 12 weeks, which is at the same slice position as (**a**), shows moderate intensity in the liver region. (**c**) The anatomical image of the same HFD Cab medaka at 17 weeks shows the liver section at the centre bottom. (**d**) The MRI-PDFF map of the HFD Cab medaka at 17 weeks, which is at the same slice position as (**b**), shows high intensity in the liver region. (**e**) The individual change in the mean MRI-PDFF shows a difference from the mean progression of the HFD group. (**f**) The HE-stained histological section of the liver of the same HFD Cab medaka at 17 weeks shows high fat accumulation. Scale bars represent 100 μm. The MR microscopy images were bicubically interpolated to have an isovoxel (34 μm).

In the HFD Cab medaka at 12 weeks, a large liver and a gall bladder were observed at the centre of a 2D signal amplitude image with TE 1.150 msec, which corresponds to a sagittal section slightly tilted around the anteroposterior axis (Fig. 5a). A heterogeneous intensity distribution was found in the liver (Fig. 5a). The distribution of MRI-PDFF in the liver was also heterogeneous, although its heterogeneity did not completely match that of the signal-amplitude image (Fig. 5a,b). The mean MRI-PDFF of the liver of the HFD Cab medaka at 12 weeks was calculated as 36.0%. After an HFD period of 17 weeks, the body size of the HFD Cab medaka did not change substantially from that at 12 weeks. A liver with a high-intensity region at the centre was observed just below an oesophagus and a digestive tract in a 2D signal amplitude image with TE 1.150 msec (Fig. 5c). The mean MRI-PDFF of the liver was calculated as 58.3% (Fig. 5d). The liver showed high MRI-PDFF in the entire region, although slight heterogeneity was observed (Fig. 5d). To investigate the individual contribution to the group trends, we plotted the individual mean MRI-PDFF values in Fig. 5e, which depicts the two mean MRI-PDFFs of the twice uneventfully recovered HFD Cab medaka at two time points of the HFD period as a connected line (Fig. 5e). The MRI-PDFF of the HFD Cab medaka showed a large increase (Fig. 5e). Just after the MR microscopy experiment at 17 weeks, we histologically analysed liver sections of the HFD Cab medaka and found a high accumulation of fat in the liver (Fig. 5f). This finding supports an increase in the fat accumulation in the liver over the HFD period.

In addition to the HFD Cab medaka, we could follow the progression of steatosis of one HFD *P53* knockout medaka during the HFD period from 19 weeks (without the overnight acclimation) to 22 weeks (with the overnight acclimation). This HFD *P53* knockout medaka lived for an additional two months.

## Discussion

In this study, we developed *in vivo* MR microscopy for a disease model in medaka by combining the hypothermic anaesthesia procedure and the high-field MR microscope. We demonstrated that the *in vivo* MR microscopy could follow and evaluate the individual progression of steatosis in a non-invasive manner using a novel imaging biomarker, MRI-PDFF. First, we demonstrated that the hypothermic anaesthesia stopped opercular movements not but heartbeats. Moreover, this state could be sustained for approximately one hour. This discovery leads to the possibility for medaka to be taken from a water container during the imaging time. Thus, the utilization of the hypothermic anaesthesia enabled us to perform high-resolution imaging using an MR microscope with enough imaging time to achieve a normal MRI experimental condition. Second, we demonstrated that feeding an HFD to wild-type Cab medaka induced fat accumulation in the liver, which resulted in the creation of an NAFLD model in medaka. Third, we applied the hypothermic anaesthesia procedure to the disease model in the MR microscopy experiment and demonstrated that the addition of an overnight acclimation period increased the proportion of uneventful recoveries of the disease model to 1. In addition, the small temperature dependence of the heartbeats after the cessation of opercular movements could prevent the medaka from waking due to the temperature increase during the imaging time. Because the uneventfully recovered medaka appeared to regain the well-being of naïve medaka, the proportion of 1 could make it possible to follow an individual disease model for more than 2 time points. This finding shows that the hypothermic anaesthesia procedure will be useful in medical and pharmacological research using various disease models in medaka. Finally, we showed high-resolution MR microscopy images of the control and HFD Cab medaka. We demonstrated that the MRI-PDFF of the HFD Cab medaka were significantly higher than that of the control Cab medaka and that the individual change did not exactly equal the group change. This finding corresponds to traditional histological results. With the *in vivo* MR microscopy, we can follow individual changes in a disease model, such as disease progression and drug administration effects. These individual changes will show a broad distribution; follow-up analyses may be useful for precision medicine. Using this approach, we can reduce the number of disease models used, which is important for both research effectiveness and animal welfare. Therefore, the *in vivo* MR microscopy expands the possibilities of using medaka disease models in medical and pharmacological research.

Although our hypothermic anaesthesia procedure may be a sufficient condition, it may not be a necessary condition for the proportion of uneventful recoveries of the disease model to become 1. We could not predict whether the transparent medaka would recover during the hypothermic anaesthesia experiment. To optimize the temperature protocol before and after the cessation of opercular movements, we could include the quantification of heartbeat strength, which corresponds to the monitoring of blood pressure during surgery. Moreover, the mechanism underlying hypothermic anaesthesia, during which a fish can sustain its life without opercular movements for an hour, needs further investigation, although lowering the temperature is known to reduce metabolic activity^19^. During the acclimation periods in our hypothermic anaesthesia procedure, the medaka could reduce their metabolic activity.

Another important animal model in fish for biological and medical research is zebrafish. Because zebrafish are tropical, the *in vivo* MR microscopy cannot be performed in exactly the same way. However, water temperature adjustments in the hypothermic anaesthesia procedure can make the *in vivo* MR microscopy applicable to zebrafish research because the hypothermic anaesthesia is a common phenomenon in fish^10^. In addition, the temperature modification of the *in vivo* MR microscopy will allow us to apply this method to aquaculture research.

The imaging procedure of the MR microscope and MRI-PDFF analysis that we used in this study may not be optimal in this situation. We could apply a faster image data-acquisition procedure^20^,^21^ to reduce motion artefacts and a more sophisticated MRI-PDFF analysis^22^,^23^,^24^ to account for high magnetic field effects and the 3D structure of the liver. However, state-of-the-art MRI techniques^20^,^21^,^22^,^23^,^24^ can be utilized to improve the accuracy of the image analysis and to extract more information from an individual disease model because the imaging conditions without a water pool are the same as those for MRI experimental conditions in general.

In summary, we demonstrate that *in vivo* MR microscopy with hypothermic anaesthesia can be used to follow and evaluate individual changes in an NAFLD disease model in medaka. This method will expand the possibilities of a human disease model in fish.

## Methods

### Ethics statement

Hypothermic anaesthesia was used for medaka. All procedures were in accordance with national guidelines and the Regulation on Animal Experimentation at Kyoto University.

### Breeding of medaka

Except for transparent medaka, adult medaka (Kyoto-cab strains and *P53*^*E241X/E241X*^ strains) were maintained in a water-circulating aquarium at 26 ± 1 °C. The photoperiod of the aquarium consisted of 15 hours of light and 9 hours of dark.

Adult transparent medaka (STIII)^12^ were maintained in a water tank. The temperature of the tank water was controlled through an air-conditioned room temperature of 26 °C. The surface of the tank water was cleaned every day. The tank water was changed once every two weeks or once a month depending on the water condition. The photoperiod of the tank was the same as that of the water-circulating aquarium.

### Hypothermic anaesthesia experiment

The heartbeats and opercular movements of naïve transparent medaka (STIII)^12^ were monitored. The medaka were fasted for 24 hours before the experiment. We placed the medaka in a small transparent tube to restrict their swimming movements. The oxygen supply to the medaka was controlled by variable water flow (6.0 ml/min; Pump III (Model 3386), Control Company, Friendswood, Texas, USA) into the tube. The tube was immersed in a water reservoir, the temperature of which was controlled by a constant water flow (120 ml/min) from a circulating chiller (COOLMAN-PAL (Model C-307), Shibata Scientific Technology Ltd., Soka, Japan). We videotaped the medaka from the side of the reservoir. We changed the temperature of the reservoir water and the oxygen supply water from the breeding water temperature (26 °C) at the mean cooling rate (−0.25 °C/min). The water temperature was measured with a thermocouple (Thermocouple type K, TC-08, Pico Technology, Cambridgeshire, UK). After opercular movements ceased, we confirmed that the medaka did not react to external stimuli. Then, we stopped cooling and oxygen supply and maintained or slightly changed the reservoir water temperature. When draining the water, we took the tube out of the reservoir and let the water flow out of the tube by opening the tube and then replacing the tube in the reservoir. After 50 or 60 minutes of anaesthesia, we returned the medaka to a small water tank containing 350 ml of the breeding water, the temperature of which was adjusted to that of the tube just before the return. Then, we let the water temperature increase to the room temperature. We counted the heartbeats and the breathing rate on a recorded video by eye. We used a total of 10 transparent medaka (1 for 20 minutes of stage 3, 9 for 60 minutes of stage 3).

### Generation of the NAFLD model in medaka

The NAFLD model in medaka was generated from medaka of the Kyoto-cab strain, a sub-strain of Cab, and from *P53* knockout medaka (*P53*^*E241X/E241X*^, created from the Kyoto-cab strain)^2^. We fed a high-fat diet (HFD32, CLEA Japan, Inc., Tokyo, Japan) to the NAFLD model medaka 3 times a day from age of 11 weeks. During each feeding time, an HFD was fed such that it was consumed by an HFD group within several minutes. In addition to an HFD, the fish were fed a normal diet (Otohime B1, Marubeni Nisshin Feed Co., Ltd., Tokyo, Japan) once a day from the age of 23 weeks. At the end of the day, we cleaned the remaining feed and changed approximately half of the breeding water to keep the aquarium clean. The control medaka were fed only the normal diet twice a day. Taking natural death into account, we prepared more than 8 medaka for each group in order to have statistically sufficient numbers (6 medaka) for each HFD and control period.

### Histological sections of medaka livers

The medaka were fasted starting the day before chemical fixation to reduce their digestive function. The medaka was deeply anaesthetized with iced water and then sacrificed. At first, the medaka were chemically fixed with Davidson’s fluid (Davidson’s fluid 50 ml: 99.5% ethanol 16 ml, 10% formalin 11 ml, acetic acid 6 ml, distilled water 17 ml)^25^. After 6 hours of chemical fixation with Davidson’s fluid at 4 °C, the fixative solution was changed to 10% formalin. The livers of the medaka were extirpated just before sectioning. Liver sections approximately 3 μm thick were obtained from three positions 100 μm apart along the short axis of the liver. Two consecutive sections were made from one liver position, and six sections were collected in total. Three sections from different positions were used for haematoxylin-eosin staining and the other three sections were used for azan staining.

### Hypothermic anaesthesia procedure in the MR microscopy experiment

Each medaka in a small water tank containing 300 ml of the breeding water was brought into a cooled MR microscope room. At that moment, the room temperature in the MR microscope room was adjusted to 18 °C, and the bore of the MR microscope was cooled by refrigerated air at 3 °C. With overnight acclimation, on the day before the MR microscopy experiment, the medaka were acclimated from the breeding water temperature (26 °C) to 18 °C overnight. Without the overnight acclimation, all medaka were brought together in the MR microscope room before the experiments started. Cooling from 26 °C to 18 °C was performed over the course of a few hours in the MR microscope room (18 °C). Just before the MR microscopy experiment, the medaka in the small water tank were cooled to 7 °C within 40 to 60 minutes. The water temperature was monitored by the thermocouple. Then, the medaka were immersed in iced water. After their opercular movements ceased, the medaka were removed from the iced water and placed in a sample tube of the MR microscope. Before placement, we wiped out the large water droplets. The sample tube was inserted into a cooled magnetic bore of the MR microscope, in which the temperature near the sample tube was kept below 18 °C during the MR microscopy experiment. The bore was cooled from both sides by refrigerated air of 3 °C and 14 °C. Just after the imaging, the medaka were placed in the breeding water, which was used for the final acclimation, at approximately 10 °C to recover from the hypothermic anaesthesia. The medaka were acclimated to the breeding temperature of 26 °C overnight by letting the water warm to 26 °C.

### The MR Microscope

We developed an MR Microscope using a 14.1 T horizontal FRP (fiber reinforced plastics) bore superconducting magnet (JMTB-14T/48/SS, Japan Superconducting Technology, Inc., Kobe, Japan) and a 600 MHz NMR spectrometer (PROT600MR, Thamway CO., LTD, Fuji, Japan) equipped with fast AD converters (FADC-12-210A, Micro Design Inc., Fuji, Japan)^8^. Triaxial gradient coils on biplane Peek bobbins produced 4.1 T/m, 3.0 T/m and 3.8 T/m at maximum along three orthogonal directions in the centre part of an imaging volume, which was calibrated with spin echo images of a spherical glass lens with a 3 mm diameter (Natsume Optical Corporation, Iida, Japan). The sample space was a PTFE (polytetrafluoroethylene) tube with a 5 mm inner diameter. To prevent the medaka from drying, the sample space was sealed with a PTFE rod and a sealing tape. Volume cross coils were used as for the radio frequency coils. The receiver coil was a 6 mm long solenoid-type coil wound on the sample space. The transmitter coil was a 15 mm long saddle-type coil. Passive decoupling was used. To reduce the magnetic field inhomogeneity, the sample space and bobbins for the radio frequency coils were made of PTFE.

### MR parameters

A gradient echo sequence with rewinder gradients was applied to obtain MR microscopic three-dimensional images with an image size of 256 × 84 × 84 and a voxel size was 34 μm × 58 μm × 58 μm. To enhance the signal-to-noise ratio on a peripheral region of the K-space, we changed the signal gain in a step-wise manner^26^. Three MR images were sequentially obtained for each medaka, in which the TEs were 1.150 msec, 1.309 msec and 1.467 msec, the repetition time (TR) was 125 msec, and the flip angle (FA) was 20°. Shorter TEs, a longer TR and a small FA were utilized to reduce the effects of *T*_1_ and *T*_2_. A strong gradient field strength (band width: 1602.7 Hz) was used to reduce chemical shift artefacts and magnetic field inhomogeneity. Shimming was not applied. The imaging time for a single three-dimensional image was 14.5 minutes, and the total imaging time was approximately 70 minutes, including the parameter-adjustment time.

### MRI-PDFF mapping

A voxel-independent IDEAL^27^ was applied to obtain a water-fraction image, a fat-fraction image and a field inhomogeneity map from three MR images with different TEs using an in-house programme. MRI-PDFF was calculated as the fat portion in the total proton density images. By taking an NMR spectrum, we assigned a fat NMR frequency shift from a water NMR frequency in each medaka, which was typically 3.607 ppm.

### Evaluation of MRI-PDFF of the liver of medaka

To analyse an isovoxel image (34 μm), we adjusted the size of the three-dimensional MRI-PDFF image from 256 × 84 × 84 to 256 × 142 × 142 using a bicubic interpolation algorithm. From the central part of the adjusted MRI-PDFF image, we selected a two-dimensional slice whose direction was closer to that of a sagittal plane. The slice selection was conducted to prevent miscalculated voxels and large blood vessels in a liver region. In the selected slice, the liver section to be analysed was manually extracted only from the inside of the liver to exclude fat tissue on the liver surface. A mean value of the liver section was evaluated as the MRI-PDFF of the medaka liver.

### Code availability

The in-house programme used to determine the voxel-independent IDEAL^27^ is available upon request to the corresponding author.

### Statistical analyses

A one-sided Fisher’s exact test and a two-sided Mann-Whitney test were used for the analyses of the recovery proportions and of the mean MRI-PDFF, respectively. Statistical calculations were performed with Microsoft Excel and the Mann-Whitney U test table. We did not perform randomization or blinding.

## Additional Information

**How to cite this article**: Ueno, T. *et al. In vivo* Magnetic Resonance Microscopy and Hypothermic Anaesthesia of a Disease Model in Medaka. *Sci. Rep.* **6**, 27188; doi: 10.1038/srep27188 (2016).

## Figures and Tables

**Table 1:** Proportion of uneventfully recovered medaka after the MR microscopy experiment with and without overnight acclimation.

	Without overnight acclimation	With overnight acclimation
Control Cab medaka	18/18 (6 w, 12 w, 16 w: 6/6)	–
HFD Cab medaka	4/13 (6 w: 1/6; 12 w: 3/6; 17 w: 0/1)	3/3 (16 w: 3/3)
Control *P53* knockout medaka	3/6 (12 w: 3/6)	–
HFD *P53* knockout medaka	6/11 (12 w: 3/6; 17 w: 0/1; 19 w: 3/4)	3/3 (22 w: 3/3)

(In the parentheses, a proportion for each feeding period is shown).

## References

[b1] Wittbrodt J., Shima A. & Schartl. M. Medaka – a model organism from the far East. Nat. Rev. Genet. 3, 53–64 (2002). 11823791 10.1038/nrg704

[b2] Taniguchi Y. . Generation of medaka gene knockout models by target-selected mutagenesis. Genome Biol. 7, R116 (2006). 17156454 10.1186/gb-2006-7-12-r116 PMC1794429

[b3] Uemura N. . Viable Neuronopathic Gaucher Disease Model in Medaka (*Oryzias latipes*) Displays Axonal Accumulation of Alpha-Synuclein. PLoS Genet. 11(4), e1005065 (2015). 25835295 10.1371/journal.pgen.1005065 PMC4383526

[b4] Matsumoto T. . Medaka as a model for human nonalcoholic steatohepatitis. Dis. Model. Mech. 3, 431–440 (2010). 20371730 10.1242/dmm.002311

[b5] Dixon W. T. Simple proton spectroscopic imaging. Radiology 153, 189–194 (1998). 6089263 10.1148/radiology.153.1.6089263

[b6] Reeder S. B., Hu H. H. & Sirlin C. B. Proton density fat-fraction: a standardized MR-based biomarker of tissue fat concentration. J. Magn. Reson. Imaging 36, 1011–1014 (2012). 22777847 10.1002/jmri.23741 PMC4779595

[b7] Noureddin M. . Utility of Magnetic Resonance Imaging Versus Histology for Quantifying Changes in Liver Fat in Nonalcoholic Fatty Liver Disease Trials. Hepatology 58, 1930–1940 (2013). 23696515 10.1002/hep.26455 PMC4819962

[b8] Ueno T. . 3D Visualization of the Medaka by Mr microscopy. The papers of technical meeting on medical and biological engineering, IEE Japan 2011(39): the 3^rd^ Biomedical Engineering International conference (BMEiCON2010), Kyoto, Japan, MBE-11-081. Tokyo, Japan: the Institute of Electrical Engineers of Japan (2011, March 31).

[b9] Kabli S. . *In Vivo* Magnetic Resonance Imaging to Detect Maglinant Melanoma in Adult Zebrafish. Zebrafish 7, 143–148 (2010). 20515295 10.1089/zeb.2009.0649

[b10] McFarland W. N. & Klontz G. W. Anesthesia in fishes. Fed. Proc. 28, 1535–1540 (1969). 4894939

[b11] Bell G. R. An Outline of Anesthetics and Anesthesia for Salmonids, a Guide for Fish Culturists in British Columbia. Can. Tech. Rep. Fish. Aquat. Sci. No. 1534, 1–16 (1987).

[b12] Wakamatsu Y., Pristyazhnyuk S., Kinoshita M., Tanaka M. & Oszato K. The see-through medaka: A fish model that is transparent throughout life. Proc. Natl. Acad. Sci. USA 98, 10046–10050 (2001). 11526229 10.1073/pnas.181204298 PMC56912

[b13] Iwama G. K., McGeer J. C. & Pawluk M. P. The effects of five fish anaesthetics on acid-base balance, hematocrit, cortisol and adrenaline in rainbow trout. Can. J. Zool. 67, 2065–2073 (1989).

[b14] Crozier W. J. & Stier T. B. Critical Thermal Incements for Rhythmic Respiratory Movements of Insects. J. Gen. Physiol. 7, 429–447 (1924). 19872148 10.1085/jgp.7.3.429 PMC2140706

[b15] Crozier W. J. & Stier T. B. On the Modification of Temperature Characteristics. J. Gen. Physiol. 7, 547–559 (1926). 19872275 10.1085/jgp.9.4.547 PMC2140844

[b16] Barcroft J. & Izquierdo J. J. The Relation of Temperature to the Pulse Rate of the Frog. J. Physiol. 71, 145–155 (1931). 16994166 10.1113/jphysiol.1931.sp002722 PMC1403056

[b17] Meuwis A. L. & Heuts M. J. Temperature Dependence of Beathing Rate in Carp. Biol. Bull. 112, 97–107 (1951).

[b18] Kleiner D. E. . Design and Validation of a Histological Scoring System for Nonalcoholic Fatty Liver Disease. Hepatology 41, 1313–1321 (2005). 15915461 10.1002/hep.20701

[b19] Ege R. & Krogh A. On the relation between the temperature and the respiratory exchange in fishes. Int. Rev. Gesamten Hydrobiol. Hydrog. 7(1), 48–55 (1914).

[b20] Lustig M., Donoho D. & Pauly J. M. Sparse MRI: The Application of Compressed sensing for Rapid MR Imaging. Magn. Reson. Med. 58, 1182–1195 (2007). 17969013 10.1002/mrm.21391

[b21] Ma D. . Magnetic resonance fingerprinting. Nature 495, 187–192 (2013). 23486058 10.1038/nature11971 PMC3602925

[b22] Jacob M. & Sutton B. P. Algebraic Decomposition of Fat and Water in MRI. IEEE Trans. Med. Imaging 28, 173–184 (2009). 19188106 10.1109/TMI.2008.927344

[b23] Hernando D., Kellman P., Haldar J. P. & Liang Z.-P. Robust Water/Fat Separation in the Presence of Large Field Inhomogeneities Using a Graph Cut Algorithm. Magn. Reson. Med. 63, 79–90 (2010). 19859956 10.1002/mrm.22177 PMC3414226

[b24] Narayan S. N. . Fast Lipid and Water Levels by Extraction With Spatial Smoothing (FLAWLESS): Three-Dimensional Volume Fat/Water Separation at 7 Tesla. J. Magn. Reson. Imaging 33, 1464–1473 (2011). 21591017 10.1002/jmri.22525 PMC3285449

[b25] Latendresse J. R., Warbrittion A. R., Jonassen H. & Creasy D. M. Fixation of Testes and Eyes Using a Modified Davidson’s Fluid: Comparison with Bouin’s Fluid and Conventional Davidson’s Fluid. Toxicol. Pathol. 30, 524–533 (2002). 12187944 10.1080/01926230290105721

[b26] Behin R., Bishop J. & Henkelman R. M. Dynamic Range Requirements for MRI. Concepts Magn. Reson. Part B (Magn. Reson. Eng.) 26B, 28–35 (2005).

[b27] Reeder S. B. . Multicoil Dixon Chemical Species Separation with an Iterative Least-Squares Estimation Method. Magn. Reson. Med. 51, 35–45 (2004). 14705043 10.1002/mrm.10675

